# Preserving Cocaine Solutions

**Published:** 1886-03

**Authors:** 


### Preserving Cocaine Solutions.

In a paper recently published in the *Droguisten Zeitung* (and *Pharm. Z.*) it is recommended to prepare stable solutions of cocaine by sterilizing them. Several methods are suggested.

1. Weigh the hydrochlorate of cocaine upon a watch-glass and place this, together with the requisite vials, filter, small glass-funnel, and all other implements which the solution will afterwards come in contact with, into a drying oven, where a temperature of 100° to 110° C. (212°–230° F.) is maintained for several hours. The requisite water is also heated for a like period. The solution is then prepared, filtered while still as hot as possible, and the vials immediately stoppered with tight plugs of pure cotton.

2. In a graduated cylinder weigh the requisite amount of hot water, add the weighed cocaine salt, and after the volume has been marked, add as much more water as will evaporate in the drying oven or on the water-bath in a few hours (about half of the volume of the solution). Then heat on the water-bath until the excess is evaporated and the former level (or the original weight) has been reached. The solution, if clear, may be at once transferred to vials which are stoppered with plugs of cotton previously sterilized by heating at 100°–110° C. Since hydrochloate of cocaine requires a heat of 185° C. (365° F.) to melt, and does not even decompose then, there can be no harm in sterilizing its solutions at the above-named temperatures.

NOTE BY ED. *Am. Drugg.*—Among the substances mostly recommended as preservatives for cocaine solutions are salicylic and boric acids, carbolic acid, thymol, and camphor. Of these salicylic and particularly carbolic acid have been found, by experience, to be irritating in many cases. Thymol appears to be better in this respect, but boric acid or camphor are unexceptionable. Of boric acid a cold saturated and filtered aqueous solution, or one containing as much as 10 grains per fluid ounce, may be used. Camphor is used in the form of camphor water. When cocaine solution is to be injected hypodermically, it is preferable to omit the boric acid ; in fact, it is always best, in this case, to use a freshly prepared solution containing nothing but the alkaloidal salt and distilled water. But if it is necessary to keep a solution for some time, probably the best way is to add to it a few drops of *chloroform*. This will prevent the development of fungi and will not act as an irritant or antagonistically to the drug. The proper proportions are :

Cocaine Hydrochlorate. . 4 parts
Distilled Water, to make 100 "
Purified Chloroform, 2 minims for
each fluid ounce.

